# Municipal wastewater treatment by the bioaugmentation of *Bacillus sp*. K5 within a sequencing batch reactor

**DOI:** 10.1371/journal.pone.0178837

**Published:** 2017-06-08

**Authors:** Yunlong Yang, Linxiang Xie, Xin Tao, Kaihui Hu, Shaobin Huang

**Affiliations:** 1 College of Life Science, Fujian Agriculture and Forestry University, Fuzhou, Fujian, CHINA; 2 The Key Laboratory of Environmental Protection and Eco-Remediation of Guangdong Regular Higher Education Institutions, Guangzhou, Guangdong, CHINA; 3 School of Environment and Energy, South China University of Technology, Guangzhou Guangdong, CHINA; The Education University of Hong Kong, HONG KONG

## Abstract

Artificial municipal wastewater was treated successfully by the bioaugmentation of *Bacillus sp*. K5 capable of simultaneous nitrification and denitrification (SND) within a sequencing batch reactor (SBR). During the long-term operation, the bioaugmentation system exhibited an excellent and steady COD and NH_4_^+^-N removal without nitrite and nitrate accumulation. The average removal efficiency for COD and NH_4_^+^-N achieved to 98% and 95%, respectively. PCR-DGGE, SEM and FISH revealed that the introduced *Bacillus sp*. K5 should be an important functional strain and exerted a critical influence on the structure of microbial community. qPCR analysis indicated that the strain K5 facilitated aerobic nutrients removal capabilities and SND might be the primary pathway for the nitrogen removal in the SBR. Overall, the SBR system used in our study should be very promising for the future wastewater treatment.

## Introduction

Nitrogen has become a key factor leading to the eutrophication of receiving waters. Accordingly, in order to reduce its discharge, increasingly stringent environmental regulations are carried out, under which circumstance it is urgent to create a novel technological solution to improve nitrogen removal. As a fact, many solutions such as physical, chemical and biological methods have been applied widely, among which biological methods seem to be more promising for some advantages. It is well known that conventional nitrogen removal is composed of two steps: (1) nitrification by autotrophs under aerobic conditions and (2) denitrification by heterotrophs under anaerobic conditions. As such, this technology usually requires extra facilities and land, resulting in high costs of treatment. Thus, it is especially necessary to explore more cost-efficient and less land-occupied technology to remove nitrogen from wastewater. Simultaneous nitrification and denitrification (SND) might be an alternative, and a variety of microorganisms have an ability of SND like *Acinetobacter calcoaceticus*[1], *Pseudomonas stutzeri*[2], *Halomonas campisalis*[3] and *Chelatococcus daeguensis*[4]. Owing to high growth rate and capability of removing ammonium and NO_X_^-^ aerobically, these strains have a great number of advantages: (1) alkalinity produced during denitrification can partly compensate for the alkalinity consumption in nitrification, thus leading to less buffer quantity[5]; (2) nitrification and denitrification can take place in a tank simultaneously, which simplifies the operation process; (3) a good ability of acclimation. However, most studies have focused on the characteristics of single strain, whereas the effectiveness of adding these functional microorganisms to the activated sludge in a sequencing batch reactor (SBR) to enhance nitrogen removal was rarely evaluated[6].

Previously, *Bacillus sp*. has been described as a novel strain with a good performance of nitrogen removal. For example, *Bacillus sp*. *LY* was isolated from the membrane bioreactor system in which the efficiency of TN removal was up to 80%. After 24-day incubation, the removal efficiency of COD by *Bacillus sp*. *LY* was 71.7%. It also can denitrify nitrate while nitrifying[7]. The *Bacillus sp*. strain YX-6 is highly effective in removing nitrite. It could degrade the nitrite nitrogen (nitrite-N) from 10 mg/L to zero in 14 h and the nitrite-N degradation rate was approximately to 100% at the DO concentration of 5.2–5.8 mg/L[8]. However, as mentioned above, the characteristics of nitrogen removal in these studies were also conducted by the single strain. To date, inoculating *Bacillus sp*. into a SBR system to strengthen SND to promote the nitrogen removal efficiency has not been reported.

The present study aimed to determine whether the bioaugmentation of *Bacillus sp*. K5 could successfully improve nitrogen removal in a lab-scale SBR for the artificial municipal wastewater treatment. Attention was concentrated on the stability of strain K5 and its cooperation with other functional microorganisms during the long-term operation, and on revealing the possible pathway of nitrogen transformation on the basis of qPCR data. This study is of great importance to exploring the potential application of K5 and establishing an effective system for wastewater treatment.

## Materials and methods

### Media and microorganism

The seed medium (SEM) modified from Merk medium was as following (g/L): peptone, 8.6; NaCl, 6.4; sodium citrate, 1.5 and potassium nitrate, 1.5. The simultaneous nitrification and denitrification medium (SNDM) comprised (g/L): sodium citrate, 4; NH_4_Cl, 0.8; KH_2_PO_4_, 0.5; Na_2_HPO_4_·7H_2_O, 1; FeSO_4_·7H_2_O, 0.1; MgSO_4_, 0.2; trace element solution, 2mL. The trace element solution was composed of (g/L): FeSO_4_·7H_2_O, 3; H_3_BO_3_, 0.01; Na_2_MoO_4_·2H_2_O, 0.01; MnSO_4_·H_2_O, 0.02; CuSO_4_·5H_2_O, 0.01; ZnSO_4_, 0.01 and ethylene diamine tetraacetic acid (EDTA), 0.5. All media had pH maintained at 7.0–7.5 and were autoclaved at 115°C for 20 min.

The strain K5 was isolated from a bio-trickling filter used for NOx treatment in the Ruiming coal-fired power plant, which is located in Guangzhou City, Guangdong Province, China. The previous study indicated that K5 has a good performance for SND and some functional genes like nirS and nosZ for nitrogen removal have been detected (unpublished data). For short-term use, the strain K5 was stored at -20°C in 30% glycerol. For long-term use, the strain K5 was freeze-dried into powder and stored at -80°C.

### SBR device and operation strategy

**Fig 1:**
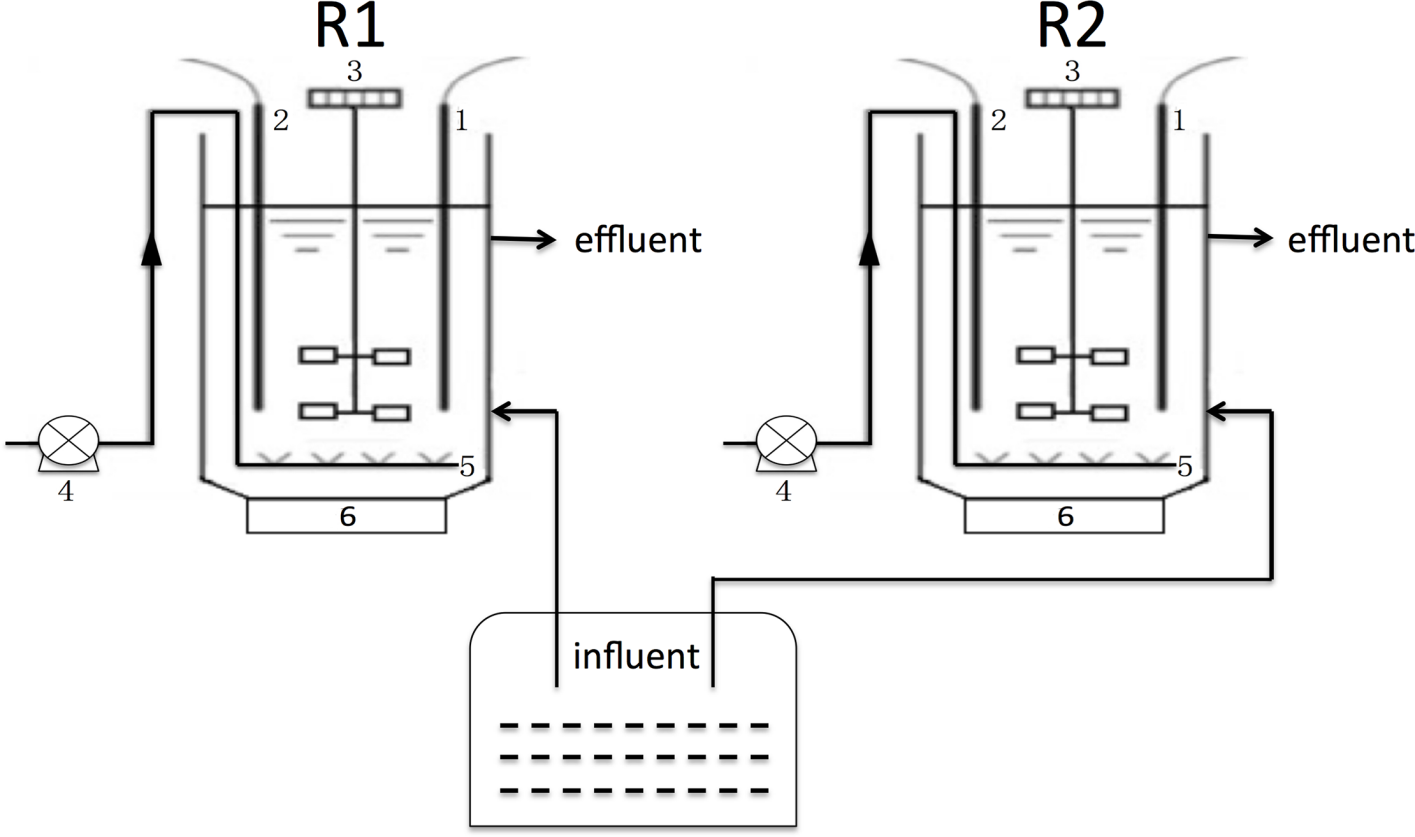
Schematic diagram of SBRs. 1. pH meter; 2. DO meter; 3. Stirrer; 4. Air pump; 5. Aerator; 6. Temperature control heater.

Two parallel SBRs with a working volume of 1.5L were used and illustrated in Fig 1. The SBR temperature was controlled at 30°C by a temperature control heater, and the airflow rate was controlled by a gas flow meter through which gas was diffused into the reactor using an aerator installed at the bottom of the SBR. The devices were operated at a cycle time of 6h, consisting of a 2-min feeding, a 240-min aerobic reaction and a 30-min settling followed by a 3-min decanting and an 85-min idle period.

Both reactors were initially inoculated with activated sludge taken from Xiangban WWTP (Fujian province, China). After the start-up, the two SBRs were operated differently: (1) R1, control SBR without K5 addition; (2) R2, bioaugmented SBR in which the pure cultures of 5% (volume ratio) were inoculated into the system every two days in order to make strain K5 predominate in SBR. Meanwhile, the volumetric exchange ratio was controlled at 30% and the dissolved oxygen (DO) was maintained at 3–4 mg/L. For long-term operation, a certain amount of sodium succinate was selected as the external carbon source to be directly added to the reactor at the beginning of every aeration period, and the volumetric exchange ratio and DO was kept at 80% and 2–3 mg/L, respectively.

### Wastewater characteristics

Simulated municipal wastewater was used, and thus no permission was required for the collection in the present study. Sodium acetate and ammonium chloride were used as carbon source and nitrogen source, respectively. In order to get more close to the real wastewater, the artificial wastewater was prepared every day with the concentrations of COD and nitrogen fluctuating in a normal range. The quality of wastewater was described in Table 1.

**Table 1:** Quality of the artificial municipal wastewater.

Item	Level	Average value
COD (mg/L)	109–221	160
NH_4_^+^-N (mg/L)	25.1–38.7	33.8
TN (mg/L)	29.5–43.2	37.1
NO_2_^-^-N (mg/L)	ND	ND
NO_3_^-^-N (mg/L)	ND	ND
pH	6.5–8.3	7.7

ND: not detected

### Microbial ecology

#### DNA extraction

The total genomic DNA of samples in SBR were extracted and purified with EasyPure Genomic DNA Kit (TransGen, China) following the manufacture’s instruction. The extracted genomic DNA was examined by 1% agarose gel electrophoresis and stored at -20°C.

#### PCR-DGGE

Polymerase chain reaction and denaturing gradient gel electrophoresis (PCR-DGGE) was conducted on the cultures in SBR. The genomic DNA, extracted as described previously, was used as template to amplify 16sRNA genes. PCR was performed in a Mastercycler gradient (Eppendorf 5331, Germany) using the following primers: GC341F (5′-CGC CCG CCG CGC CCC GCG CCC GGC CCG CCG CCC CCG CCC G CCT ACG GG A GGC AGC AG-3′) and 907R (5′-CCG TCA ATT CCT TTG AGT TT-3′). DGGE was carried out in a Universal Mutation Detection System (BIO-RAD DCodeTM, USA). The gel contained a gradient of denaturant raging from 30% to 60% (100% denaturant is 7 M urea and 40% deionized formamide). DGGE was run at 200 V for 5 h at 60°C. After electrophoresis, the gel was stained with GoldView II (Solarbio, Beijing, China) for 30 min and viewed with a UV transilluminator (BIO-RAD, Italy).

#### SEM observation

The morphology of the bacteria in active sludge was examined with an environmental scanning electron microscope (XL30, Philips, ESEM). The samples were pretreated by fixing with 2.5% pentanediol in a 0.1 M phosphate buffer, and then soaked in 1% osmic acid. Subsequently, the samples were washed and dehydrated in a graded series of ethanol solutions (50, 70, 80, 90 and 100%). The samples were dried by the critical point method and coated with gold.

#### Fluorescence in situ hybridization

The nirS R3cd primer [5′-GA(C/G)TTCGG(A/G)TG(C/G)GTCTTGA-3′][9] was chosen to be used as an oligonucleotide probe. Fluorescence-labeled oligonucleotides were synthesized by Sangon Biotech (Shanhai, China) and labeled with fluorescein isothiocyanate (FITC). The nirS fluorescence in situ hybridization (FISH) was performed according to the following protocol (1) Immobilization: activated sludge was fixed in 4% paraformaldehyde in PBS for 2h at 4°C, washed three times with PBS, and resuspended in PBS. 50 μl of fixed activated sludge was transferred onto microscope slides, dehydrated in absolute ethanol for 3 min, air-dried, washed for 15 min with 0.4%Tritonx-100 and PBS, respectively, and air-dried again. (2) Denaturation: slides were put into the well containing denaturing solution (formyl amide 70%, 2×SSC, 0.1mM EDTA), incubated for 8 min at 75°C, and then transferred to the well containing 70% ethanol precooled at -20°C, incubated for 2 min. Afterwards, slides were dehydrated three times in absolute ethanol precooled at -20°C for 2 min and air-dried. (3) Hybridization: 50 μl of hybridization buffer (0.9 M NaCl, 50 mM Na_2_HPO4, 0.1% wt/vol SDS, 0.5 mg yeast tRNA ml^−1^, 10×Denhardt’s solution and 1μl FITC-labeled probe) was pipetted onto each well and slides were incubated overnight at 42°C in humidified chambers. (4) Washing: Slides were washed three times in washing buffer (0.9 M NaCl, 50 mM Na_2_HPO4, 0.1% wt/vol SDS) for 2 min at 45°C, dehydrated with absolute ethanol and air-dried. Images were visualized with an inverted fluorescence microscope (Olymus IX71, Japan).

### Quantitative polymerase chain reaction (qPCR)

#### Primer design

Quantitative analysis was conducted on the target fragments of the following functional genes: ammonia monooxygenase (amoA), copper-containing nitrite reductase (nirK), cd1-containing nitrite reductase (nirS) and nitrous oxide reductase (nosZ). All primers synthesized by Invitrogen Biotechnology Company (Shanghai, China) are summarized in Table 2, and diluted to a concentration of 10 μmol/L.

**Table 2:** Primers of target genes used in qPCR analysis.

Target gene	Primer	Primer sequence (5’-3’)	Amplification size (bp)	Reference
*amoA*	amo598f	GAATATGTTCGCCTGATTG	120	[10]
	amo718r	CAAAGTACCACCATACGCAG		
nirK	nirK876	ATYGGCGGVAYGGCGA	163	[11]
	nirK1040	GCCTCGATCAGRTTRTGGTT		
nirS	nirScd3aF	GT(C/G)AACGT(C/G)AAGGA(A/G)AC(C/G)GG	425	[9]
	nirSR3cd	GA(C/G)TTCGG(A/G)TG(C/G)GTCTTGA		
*nosZ*	nosZ1527F	CGCTGTTCHTCGACAGYCA	250	[12]
	nosZ1773R	ATRTCGATCARCTGBTCGTT		

#### qPCR

qPCR was performed on the LightCycler480 instrument (Roche, Switzerland) in a final volume of 25 μl. The PCR reaction mixtures consisted of: 10 μl SybrGreen qPCR Master Mix (BBI, USA), 2 μl template DNA (Sample DNA or plasmid DNA for standard curves), 0.4 μl forward primers (10 μm), 0.4 μl reverse primers (10 μm) and sterile water (Milipore, USA). qPCR was carried out in a final three-step thermal cycling procedure including 3 min at 95°C, followed by 45 cycles composed of 15s at 95°C, 20s at 57°C and 30s at 72°C. After cycles, the melting curve analysis was conducted. Sterile water was used as a negative control and the data obtained from qPCR was normalized to copies per gram of dry active sludge (after settlement, the active sludge was subjected to a centrifugation of 5000rpm for 10 min and then the precipitate was dried for 24h at 60°C in a drying oven).

#### Standard curves

The plasmids containing functional genes (amoA, nirK, nirS and nosZ) were manufactured by Sangon Biotechnolgoy Company (Shanghai, China). A series of 10-fold concentrations used for qPCR standard curves were yielded by diluting standard samples. The R^2^ value for all standard curves exceeded 0.99, which showed that they presented good linear relationships over the concentration ranges used in this study.

### Analytical methods

The total nitrogen (TN), ammonium nitrogen (NH_4_^+^-N), nitrite nitrogen (NO_2_^-^-N), nitrate nitrogen (NO_3_^-^-N) and COD were analyzed using Standard Methods[13]. DO (DO-2200, Yilun, China) and pH (PHS-3E, Leici, China) were determined online.

## Results and discussion

### Performance in two parallel SBRs

**Fig 2:**
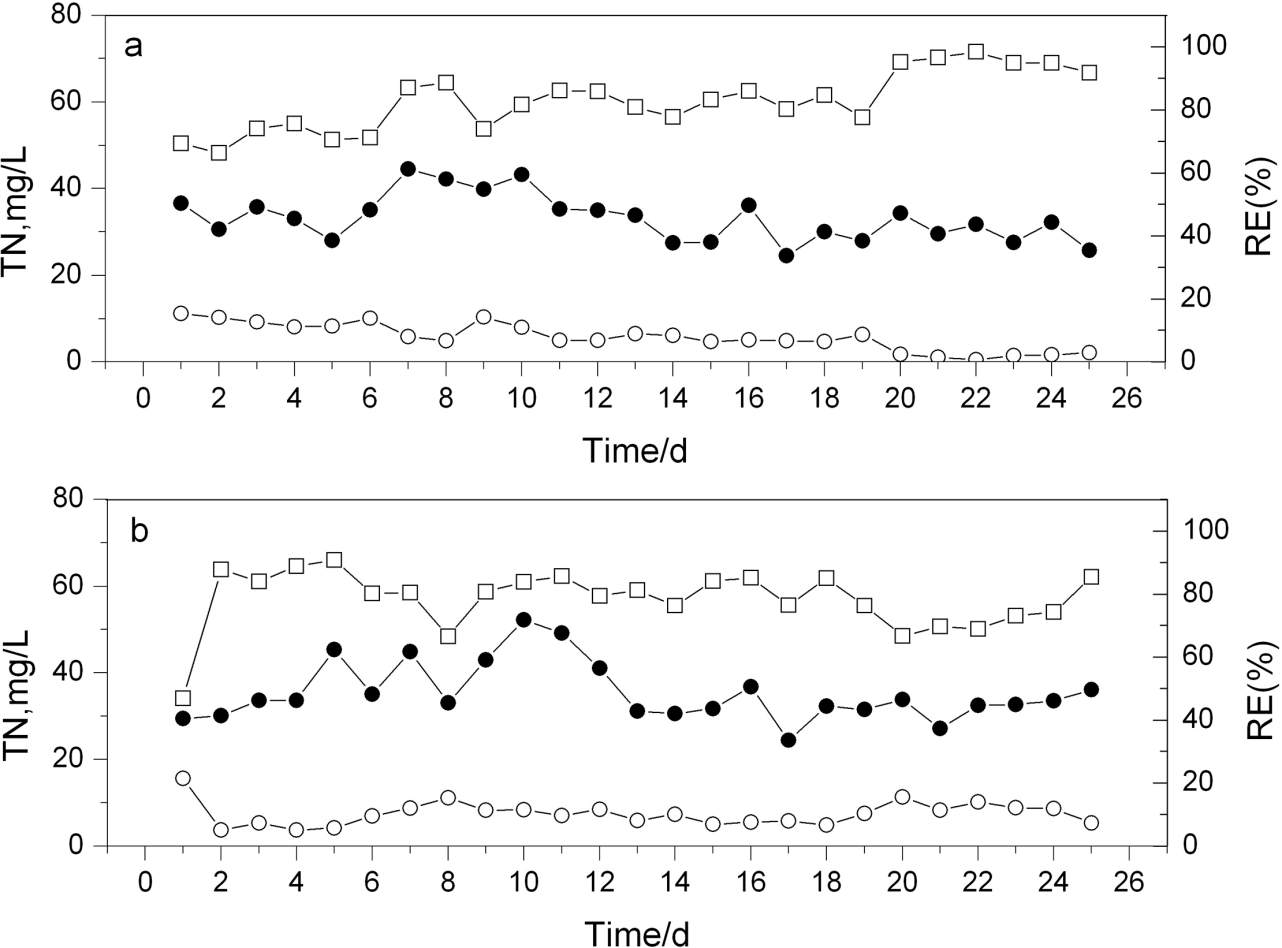
Comparison of the removal efficiency of TN in R1 (b) and R2 (a). Symbols: ● influent; ○ effluent; □ RE.

Two parallel SBRs were used to investigate the performance of bioaugmentation. It was found that there was no significant difference in COD and ammonium-N removal between the two SBRs (data not shown). However, it was remarkably different for total nitrogen (TN) removal (Fig 2). In R1 (control SBR), the average TN removal efficiency (RE) was 78.1% (Fig 2B), whereas the average RE of TN in R2 (bioaugmented SBR) achieved to 82.8% and even exceeded 90% from 20d on (Fig 2A). These results suggest that the bioaugmented SBR was more effective for TN removal, which was in good agreement with the previous report[6].

### Long-term operation in the bioaugmented SBR

**Fig 3:**
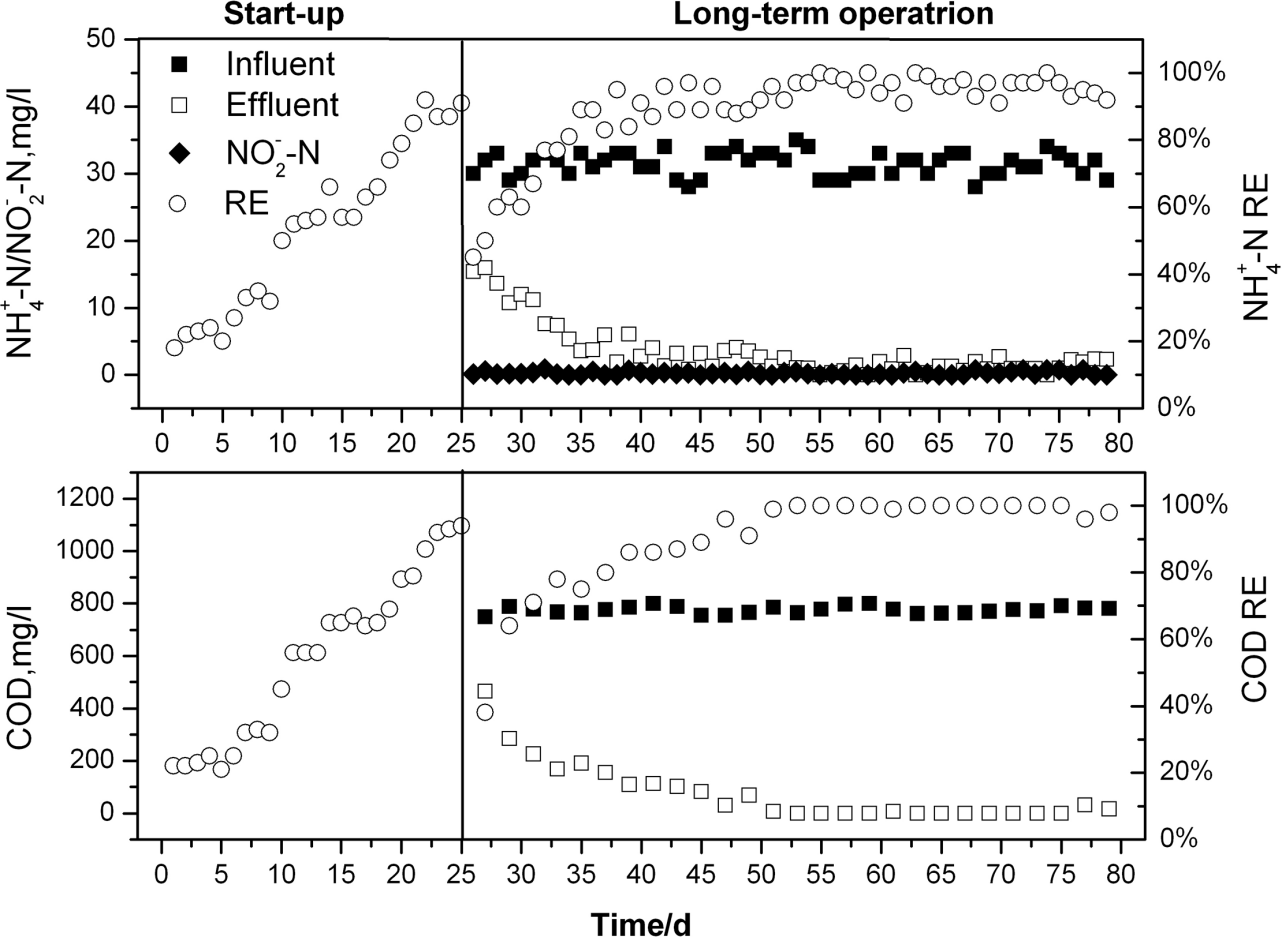
Performance of SBR for COD and NH_4_^+^-N removal from municipal wastewater.

The SBR system was started and operated for about 80 days with a relatively steady load of artificial municipal wastewater (Fig 3). During the startup stage, the SNDM was used to cultivate microorganisms, and the pure cultures of K5 (5% v/v) were inoculated to SBR every two days in order to make K5 predominate in the system. With time elapsing, the RE of NH_4_^+^-N and COD elevated progressively, and finally reached 91% and 94% on day 25, respectively, suggesting that the active sludge with a good performance for SND had formed.

From day 26 on, the artificial municipal wastewater was fed to the SBR system, and the Re of NH_4_^+^-N dropped abruptly to 50%. Afterwards, the RE increased step by step to 89% on day 35, after which RE was almost steady fluctuating from 90% to 100% during the following long-term operation. Likewise, COD also presented the similar trends. Its RE decreased rapidly from 94% on day 25 to 38% on day 26, and increased gradually to 96% on day 47. Although the RE on day 48 went down, it went up quickly and even a high level of almost 100% maintained for about 30 days.

As shown in Fig 3, the REs of both NH_4_^+^-N and COD decreased sharply on day 26, which might be caused by the sensitivity of strain K5 to the completely different nitrogen environment, since this environment was switched from SNDM to simulated wastewater. Once the bacteria adapted themselves to the system, REs reached a stable rate step by step. In addition, it could be found that both nitrite and nitrate (data not shown) were rarely accumulated, indicating that the system had a good capability for aerobic denitrification, which might be attributed to the stain K5 characteristics for nitrogen removal (unpublished data). Many reports have confirmed that simultaneous COD and nitrogen removal could be achieved aerobically in a SBR system[6, 14]. For example, in a pilot-scale SBR inoculated with PCN bacteria, the averaged effluent concentrations of COD and NH_4_^+^-N were 20.6 and 0.69 mg/L, respectively[6]. Similarly, the SBR inoculated with the strain K5 was employed in the current study, and results presented herein indicated that both COD and NH_4_^+^-N could also be removed effectively. As a result, the present system has many advantages over the conventional systems: (1) nitrification and denitrification could take place simultaneously in the aeration tank and then it is unnecessary to construct additional systems for nutrients removal; (2) the flexibility, high efficiency and good resistance capability to fluctuations could be obtained because the maximum growth rates of heterotrophic nitrifiers are five to ten times than those of autotrophic nitrifiers[6]. Consequently, the SBR system used in our study should be very promising for the future wastewater treatment.

### Stability of the strain K5 in the SBR system

**Fig 4:**
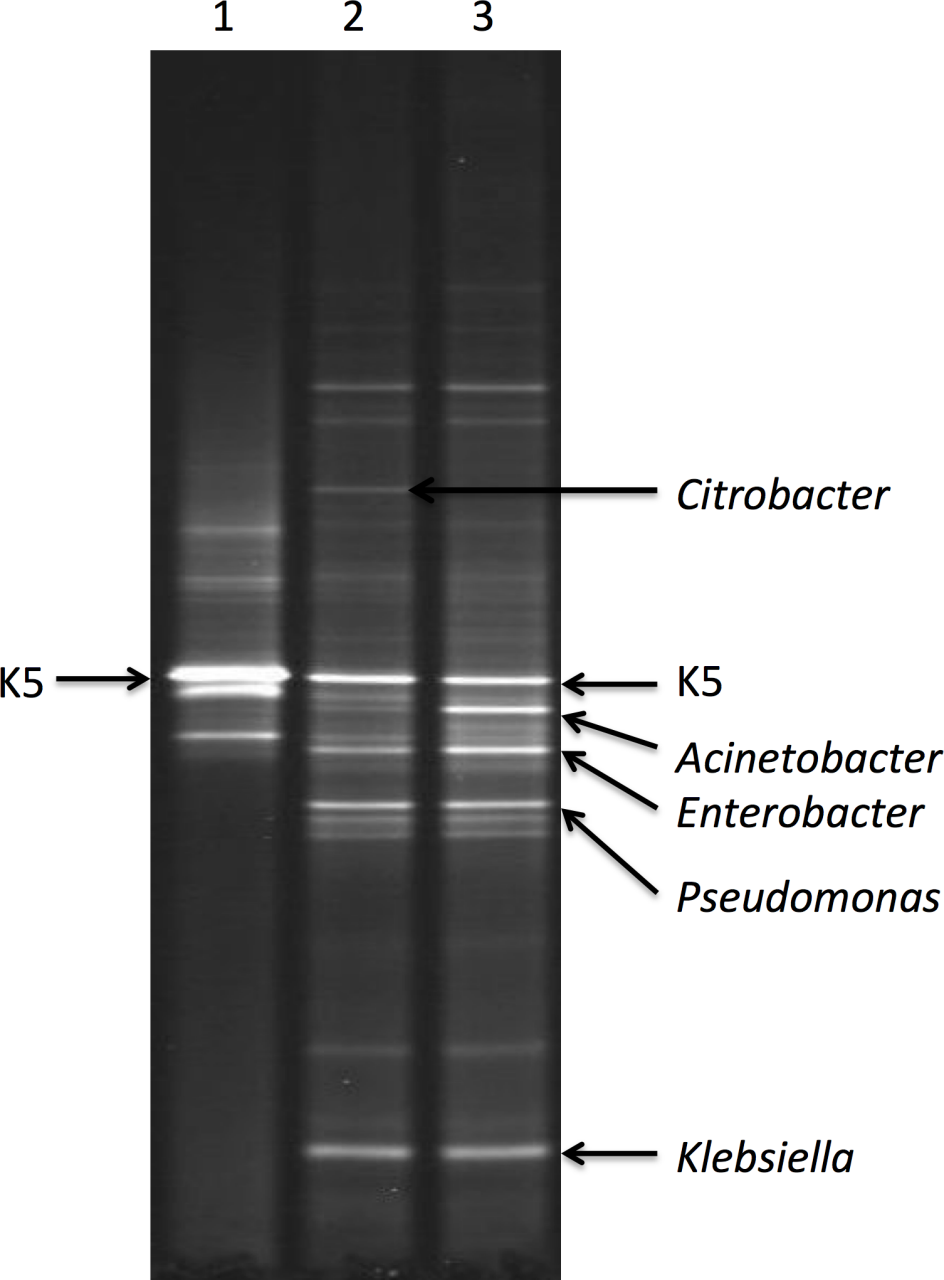
DGGE profile of PCR-amplified 16s rDNA fragment. Fragments were obtained with bacterial primer set and DNA isolated from active sludge of 15d (Lane 1), 55d (Lane 2) and 77d (Lane 3).

In order to investigate the microbial diversity, especially the stability of K5 in the system, PCR-DGGE was carried out on the active sludge of 15d, 55d and 77d, and results were shown in Fig 4. Obviously, the strain K5 had been predominating in the system, though some other genera such as *Acinetobacter*, *Enterobacter*, *Pseudomonas*, *Klebsiella* and *Citrobacter* also existed. In view of the above results (Fig 3), it was concluded that *Bacillus sp*. K5 can not only inhabit in SBR, but also cooperate well with other microorganisms to effectively remove nitrogen and COD during the long-term operation.

It is worth noting that there were no significant changes in the microbial diversity of 55d and 77d, except for stains *Acinetobacter* and *Citrobacter*. The strain *Citrobacter* became weaker, whereas the strain *Acinetobacter* grew stronger. Results suggested that these bacteria were accustomed to the wastewater environment and maintained relatively stable, which could also be confirmed from Fig 3. Additionally, how to successfully apply bioaugmentation technology to the wastewater treatment rests with the adaption of extra strains with indigenous microorganisms, which means introduced strains should survive and keep activity in the system. Most importantly, extra strains do not interfere with the function of native bacteria but rather cooperate with each other to degrade pollutants. As a result, some methods like daily repeated adding[15], embedded strategy[16] and one time addition[6] were explored to avoid the influence of native microorganisms. In this study, K5 was also successfully introduced to the active sludge and exhibited excellent stability and compatibility with the SBR ecosystem, indicating a great potential for actual application.

### Surface morphology and FISH of active sludge

**Fig 5:**
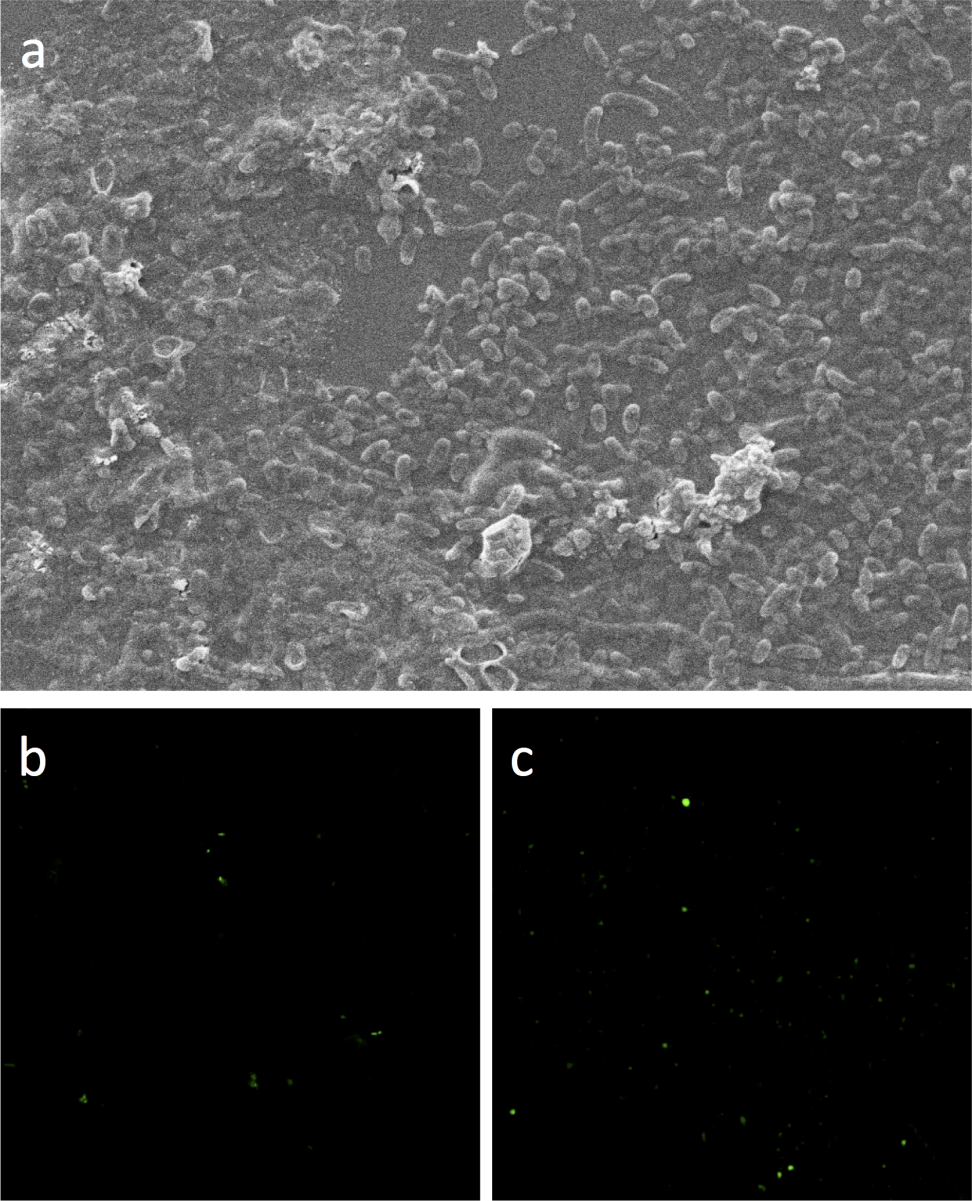
Images for SEM and FISH. (a) micrograph of microorganisms grown in the active sludge of 60d; (b) FISH-detection of nirS probe in R1; (c) FISH-detection of nirS probe in R2.

To observe the surface morphology, the active sludge sample of 60d was taken out for analysis. The surface characteristics of samples were performed by SEM, and results were shown in Fig 5A. The surface observation of active sludge revealed that it was composed of many microorganisms like bacilli and cocci bacteria, among which the rod-shaped bacteria were observed as predominant species existed in the active sludge.

In addition, as mentioned above, the nirS gene was involved in the strain K5, which could be used as a marker to evaluate K5 in the active sludge. Consequently, the nirS-FISH experiments were conducted in two parallel SBRs, and results were shown in Fig 5. Obviously, FISH-detection in R2 (bioaugmented reactor, Fig 5C) presented stronger signals than in R1 (control reactor, Fig 5B), suggesting that there were more functional microorganisms in R2. As such, in consideration of both SEM and FISH, the strain K5 should be an important functional bacterium in the bioaugmented SBR.

### Absolute abundance of functional genes

**Fig 6:**
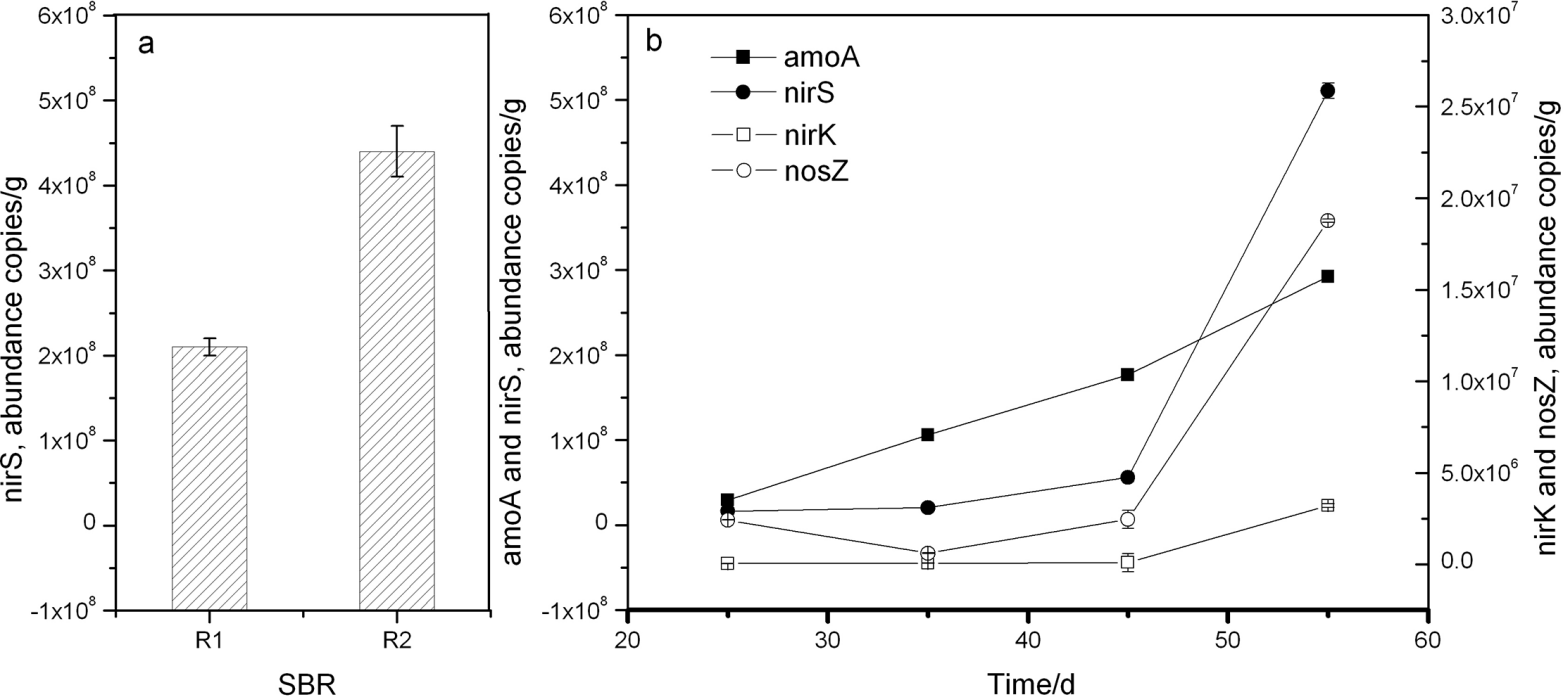
Absolute abundance of functional genes. (a) nirS abundance in two parallel SBRs on 46d; (b) amoA, nirK, nirS and nosZ abundance in the bioaugmented SBR. Error bars indicate standard deviations of three replicates. Invisible error bars indicate that standard deviations are smaller than the marker size.

As described above, the nirS gene involved in K5 plays a vital role in simultaneous nitrification and denitrification and thus was chosen as a representative gene to make a comparison between the two parallel reactors. It could be observed that the absolute abundance of nirS achieved to 4.4×10^8^ copies/g in R2, which was almost two times that in R1 (Fig 6A). Undoubtedly, this phenomenon was ascribed to the bioaugmentation of K5.

To determine the dynamic population shifts of functional genes, the absolute abundance of amoA, nirK, nirS and nosZ were also quantified during the operation period of bioaugmented SBR. Results from Fig 6B showed the absolute abundance of amoA markedly increased from 2.9×10^7^ copies/g on day 25 to 2.9×10^8^ copies/g on day 55, with 10-fold increase being obtained. In general, amoA gene, nxrA gene and anammox 16s rRNA are the three functional genes involved in the NH_4_^+^-N transformation. The ammonia monooxygenase coding gene amoA is usually regarded as the marker of aerobic ammonia oxidation by which NH_4_^+^-N is oxidized to NO_2_^-^-N[10], which is also the first and an important step in simultaneous nitrification and denitrification. The nxrA gene encodes nitrite oxidase that could oxidize NO_2_^-^-N to NO_3_^-^-N[17]. The anammox 16s rRNA gene is often used as the marker of anaerobic ammonium oxidation, in which NH_4_^+^-N and NO_2_^-^-N are converted to N_2_ under anoxic conditions[18]. However, NO_3_^-^-N was not detected in effluent and the whole operation was conducted under aerobic conditions. Therefore, both nxrA gene and anammox 16s rRNA gene were not investigated in the current study. In view of gradually increasing trend of amoA, it is concluded that the aerobic ammonia oxidation was the major NH_4_^+^-N removal pathway in this SBR system.

The nirK, nirS and nosZ genes are important functional genes involved in denitrification. Results in Fig 5 indicate they all exhibited a continuous increase, raging from 4.04×10^4^ copies/g, 1.65×10^7^ copies/g and 2.42×10^6^ copies/g on day 25 to 3.21×10^6^ copies/g, 5.11×10^8^ copies/g and 1.88×10^7^ copies/g on day 55, respectively. It has been reported that there are two functionally and physiologically equivalent types of nitrite reductases responsible for the reduction of NO_2_^-^-N to NO: a cytochrome cd1 encoded by nirS and a Cu-containing enzyme encoded by nirK[19]. The genes of nirK and nirS are usually used as nitrite reduction markers to explore the denitrifying bacterial community[20, 21]. However, the nirS gene was much more abundant than nirK during the entire operation period. This phenomenon was congruent with previous studies in which the nirS gene was reported to be environmentally more abundant than nirk gene[19, 22]. On the other hand, this result might be attributed to the strain K5, since this strain harbors the nirS gene (unpublished data). Higher nirS gene abundance plays a critical role in nitrite reduction, but it was also a primary contributor to NO greenhouse gas production. As a marker for complete denitrification, the nosZ gene serves to encode nitrous oxide reductase responsible for N_2_O to N_2_ reduction[23], which is the final step in the denitrification pathway and is conducive to control greenhouse gas emissions. Fortunately, the nosZ gene abundance in the present SBR system increased about by 10-fold (Fig 6B). This increase in the nosZ gene promoted the last step of denitrification, thus leading to not only complete denitrification but also a potential reduction in the emission of N_2_O.

The four above genes taking part in simultaneous nitrification and denitrification displayed a clear increasing trend in our SBR system. Specifically, from day 45 to day 55, they went up rapidly, which was in good agreement with the results in Fig 4 that from day 55 on, both NH_4_^+^-N and COD maintained a high level of removal efficiency.

### Nitrogen removal pathway

To explore the nitrogen removal pathway in the present SBR, the cyclic profiles of NH_4_^+^-N, NO_2_^-^-N, NO_3_^-^-N and COD on 61d were conducted, and results were exhibited in Table 3. It was observed the ammonia-N decreased rapidly from 30.1 to 0.93 mg/L during 2.5h, while COD dropped from 778 to 7.8 mg/L within 3.5h. Although nitrite-N accumulated to 0.08 mg/L at 1.5h, it was then not detected after 3.5h. In addition, there was no nitrate-N accumulation (not detected), which might be attributed to the fact that there was not sufficient nitrite to be oxidized to nitrate because of a high concentration of nitrite reductase gene copies (Fig 6).

**Table 3:** Changes of NH_4_^+^-N, NO_2_^-^-N, NO_3_^-^-N and COD in one cycle on 61d.

Time (h)	Item (mg/L)			
	NH_4_^+^-N	NO_2_^-^-N	NO_3_^-^-N	COD
0	30.1±2.1	ND	ND	778.0±10.2
1.5	9.15±0.6	0.08	ND	325.3±7.1
2.5	0.93±0.02	0.024	ND	70.2±2.0
3.5	0.95±0.06	ND	ND	7.81±0.26
4.5	0.90±0.03	ND	ND	7.78±0.27

ND: not detected

**Fig 7:**
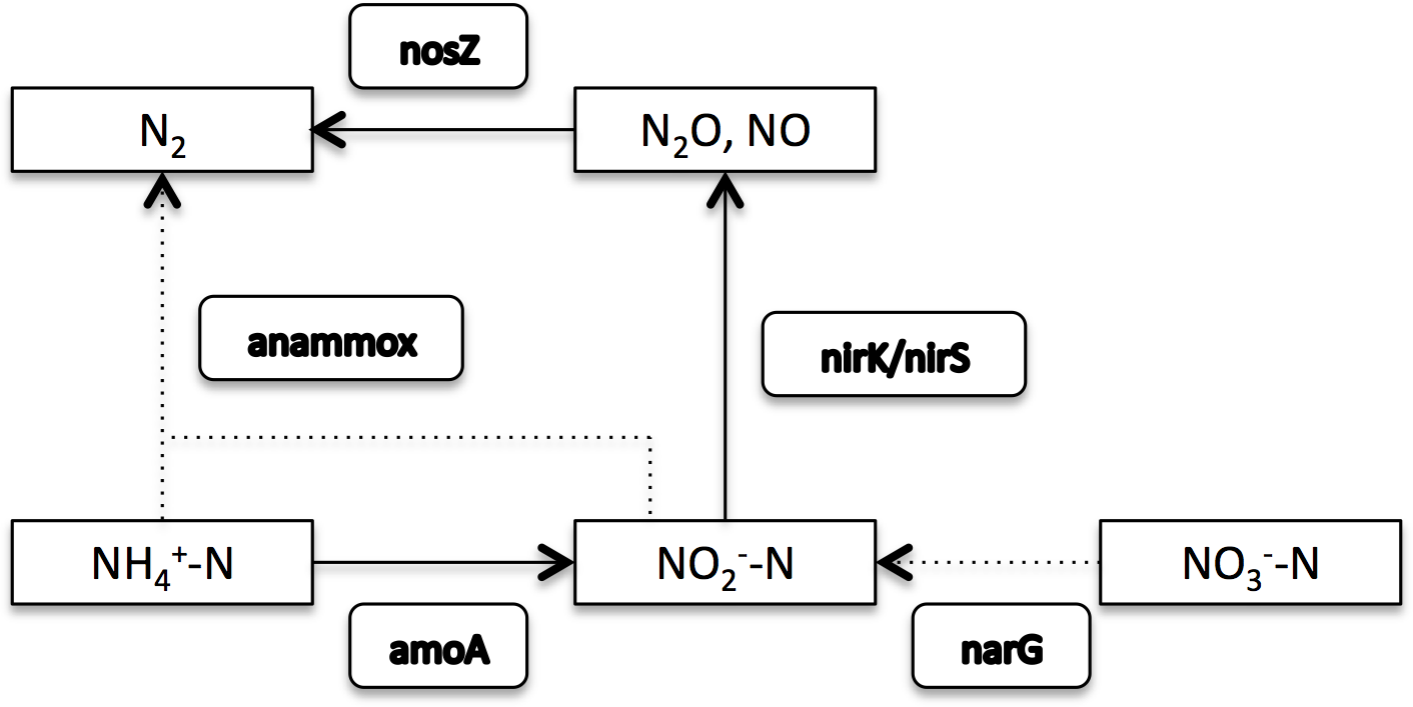
Promoted nitrogen removal pathway in the SBR system. Solid line: augmented pathway; Dotted line: weakened pathway.

Nitrite is an important intermediate in simultaneous nitrification and denitrification because a significant amount of nitrite is the premise for the subsequent denitrification, so the stability of nitrite accumulation was investigated in a sequencing batch reactor[24]. However, nitrite hardly accumulated in the present SBR system (Table 3), which was mainly ascribed to a high level of nirS copies (Fig 5). Additionally, nitrite-N was also affected by amoA that is conducive to the accumulation of nitrite. As mentioned above, the whole operations were carried out under aerobic conditions and nitrate was not detected. Therefore, based on these results, the following nitrogen removal pathway was promoted in our SBR system: ammonium was oxidized to nitrite, and then the produced nitrite was rapidly reduced to N_2_ due to the bioaugmentation of K5 (Fig 7). As far as other nitrogen transformations like anammox and reduction of nitrate to nitrite are concerned, they must exist and function since there are anoxic zones within the system, but they should play a marginal role under our conditions. In summary, simultaneous nitrification and denitrification (SND) might be the primary pathway for the nitrogen removal in this SBR system.

## Conclusions

A SBR system was used to treat the artificial municipal wastewater by the bioaugmentation of *Bacillus sp*. K5 capable of simultaneous nitrification and denitrification (SND). High nutrients removal efficiency for COD (90–100%) and NH_4_^+^-N (85–100%) were simultaneously obtained in this system during the long-term operation. The bioaugmentation made K5 predominant and strengthened the performance of SND to promote aerobic nutrients removal. The data from qPCR suggested that SND might be the primary pathway for nitrogen removal. Consequently, the bioaugmentation of *Bacillus sp*. K5 in the SBR could be regarded as an effective technology for the treatment of municipal wastewater.

## Supporting information

S1 File Data for qPCR. (XLSX)
